# Veterinary Medical Association of New York County

**Published:** 1894-03

**Authors:** J. Elmer Ryder

**Affiliations:** Secretary


### SOCIETY PROCEEDINGS.

### VETERINARY MEDICAL ASSOCIATION OF NEW YORK COUNTY.

The first regular meeting of the Veterinary Medical Association of New York County was held February 6th, 1894.

The meeting was called to order by the President, Dr. R. S. Huidekoper. Upon the roll-call the following members responded to their names, viz.: Drs. Amling, Becket, Bieser, Buckley, C. C. Catta ach, J. J Cattanach, Dickson, Delany, Ferster, Field, Giffen, Gill, Huidekoper, Jackson, Loomes, Liautard, Hickman, Hanson, Neher, O'Shea, Parkerson, Parsons, Robertson, Ryder, Richards, Sherwood, Sielman, Fergeson and Turner.

The minutes of the previous meeting were read and approved. Dr. Huidekoper then stated that the Secretary had mailed each member a list of the practitioners registered in New York County, and stated that the list contained the names of a number who have left the city, and several who have died. He asked that each member correct the list as far as he knows of errors, and return the same to the Secretary, in order that the Association may know the exact number of practitioners, and their residence in New York County.

The Committee on Constitution and By-laws submitted to the meeting the constitution and by-laws as formulated by them, after which they were read section by section, and a general discussion by the members followed. After a number of amendments were made and an extra section added they were adopted.

Moved by *Dr. Giffen*, and seconded by Dr O'Shea, that the President appoint the Judiciary Committee as adopted in the by-laws. Carried.

Moved by *Dr. Liautard*, and seconded by Dr. Hanson, that the Committee on By-laws rewrite them as amended, and read them at the next meeting Carried.

Moved by *Dr. Liautard*, and seconded by Dr. C. C. Cattanach, that a committee of three be appointed by the President to select a draft for the certificates of the Association with the wording adopted in the by-laws. Carried.

Moved by *Dr. Ferster*, and seconded by Dr. Liautard, that the Judiciary Committee consider plans for incorporation, and report at next meeting. Carried.

Moved by *Dr. Gill*, and seconded by Dr. Field, that a vote of thanks be extended to Dr. Hanson for his efforts in causing the dissolution of another society, and recommending them to join this Association. Carried.

Moved by *Dr. Gill*, and seconded by Dr. O'Shea, that the Committee on Conference be dissolved with thanks. Carried.

The President then announced the following committees: JUDICIARY, Dr. Giffen (Chairman), Drs. Buckley, Ryder, Turner, O'Shea. CERTIFICATES, Dr. Liautard (Chairman), Drs. Sherwood, Becket. Adjourned.

J. ELMER RYDER, D.V.S., Secretary.

